# Hyperphosphorylation of ribosomal protein S6 predicts unfavorable clinical survival in non-small cell lung cancer

**DOI:** 10.1186/s13046-015-0239-1

**Published:** 2015-10-21

**Authors:** Bojiang Chen, Zhi Tan, Jun Gao, Wei Wu, Lida Liu, Wei Jin, Yidan Cao, Shuang Zhao, Wen Zhang, Zhixin Qiu, Dan Liu, Xianming Mo, Weimin Li

**Affiliations:** Department of Respiratory and Critical Care Medicine, West China Hospital of Sichuan University, No. 37, Guo Xue Street, Chengdu, Sichuan 610041 China; Inspectiong and Quarantine Technical Center of Sichuan Entry-Exit Inspection and Quarantine Bureau, Chengdu, China; Department of Toxicological Inspection, Sichuan Center for Disease Prevention and Control, Chengdu, China; Department of Outpatient, West China Hospital of Sichuan University, Chengdu, China; Department of Pathology, West China Hospital of Sichuan University, Chengdu, China; Department of Respiratory Medicine, Second Affiliated Hospital, Chongqing Medical University, Chongqing, China; Laboratory of Stem Cell Biology, West China Hospital of Sichuan University, Chengdu, China

**Keywords:** Ribosomal protein S6 (rpS6), Non-small cell lung cancer (NSCLC), Hyperphosphorylation, Survival, Signaling pathway

## Abstract

**Background:**

Ribosomal protein S6 (rpS6), a component of the 40S ribosomal subunit, is involved in multiple cellular bioactivities. However, its clinicopathological significance in non-small cell lung cancer (NSCLC) is poorly understood.

**Methods:**

Expressions of total rpS6 (t-rpS6) and phosphorylated rpS6 (Ser235/236, p-rpS6) were detected immunohistochemically in 316 NSCLC tissues and 82 adjacent controls, followed by statistical evaluation of the relationship between proteins expressions and patients’ survivals to identify their prognostic values. Cytological experiments with overexpressing or silencing rpS6 by lentivirus in human bronchial epithelial (HBE) and NSCLC cell lines were performed to explore potential mechanisms by which rpS6 affects the clinical development of NSCLC. Additionally, specific RNA interference for Akt1, Akt2, Akt3, Akt inhibitor and subsequent cellular bioactivity tests were employed as well to investigate the upstream regulation of rpS6.

**Results:**

Positive rates of t-rpS6 and p-rpS6 were both significantly increased in NSCLC tissues, compared with controls (82.91 *vs* 62.20 % for t-rpS6; 52.22 *vs* 21.95 % for p-rpS6; both *P* < 0.001). However, only hyperphosphorylation of rpS6, expressed as either elevated p-rpS6 alone or the ratio of p-rpS6 to t-rpS6 (p-rpS6/t-rpS6) no less than 0.67, was greatly associated with the unfavorable survival of NSCLC patients, especially for cases at stage I (all *P* < 0.001). The independent adverse prognostic value of hyperphosphorylated rpS6 was confirmed by multivariate Cox regression analysis (hazard ratios for elevated p-rpS6 alone and p-rpS6/t-rpS6 no less than 0.67 were 2.403, 4.311 respectively, both *P* < 0.001). Overexpression or knockdown of rpS6, along with parallel alterations of p-rpS6, led to increased or decreased cells proliferations respectively, which were dependent on redistributions of cell cycles (all *P* < 0.05). Cells migration and invasion also changed with rpS6 interference (all *P* < 0.05). Furthermore, upstream overexpression or knockdown of Akt2 or Akt2 phosphorylation inhibition, rather than Akt1 or Akt3, resulted in striking hyperphosphorylation or dephosphorylation of mTOR, p70S6K and rpS6 (all *P* < 0.05), without any change in total proteins expressions. Further tests showed markedly accompanied variation of cells proliferation, cell cycle distribution and invasion (all *P* < 0.05).

**Conclusion:**

Hyperphosphorylation of rpS6, probably regulated by the Akt2/mTOR/p70S6K signaling pathway, is closely relevant to the progression of NSCLC and it might be served as a promising therapeutic target for NSCLC treatment.

**Electronic supplementary material:**

The online version of this article (doi:10.1186/s13046-015-0239-1) contains supplementary material, which is available to authorized users.

## Introduction

Lung cancer is widely considered as the leading cause of cancer-related death, attributing more than one million deaths per year around the world [1]. Non-small cell lung cancer (NSCLC) accounted for the overwhelming majority of lung cancer. Although much progress has been made in the application of multidisciplinary treatments for lung cancer, its 5-year survival rate only slightly improved from 12.3 % in 1970s to 16.9 % in 2000s [1, 2]. Such a pessimistic situation is largely ascribable to the underappreciated pathogenesis of lung cancer. Up to recent, multitudinous studies have verified the principal roles of various genetic abnormalities in the development of lung cancer, which finally appear as the dysfunctions of numerous proteins [3–5].

Ribosomes are important intracellular particles for protein synthesis with the composition of RNA and proteins. Ribosomal protein S6 (rpS6) is one of the components of the 40S ribosomal subunit. Although still not fully understood, rpS6 has been functionally regarded as the stimulator and/or inhibitor of certain types of mRNA translation, as well as the regulator of cellular metabolisms, cells size, survival and proliferation [6–8]. In most cases, phosphorylation at the evolutionary conserved c-terminal serine residues between Ser235 and Ser247 is believed to be the activation of rpS6 and exerts important successive effects [8–10]. The response of striatal to dopaminergic pharmacological manipulations, muscle fibers to the progressive resistance exercise and even the myocardial cells with the inflammatory stimulus were all related to the phosphorylation of rpS6 (p-rpS6) [10–13]. However, an opposite finding that rpS6 could combine with the U3 nucleoprotein complexes in nucleolus without the phosphorylation was also reported [14], indicating the multiple functional forms of rpS6.

Recently, much attention has been paid to the effects of rpS6 in tumors. The over expression and activation of rpS6 in lymphangioleiomyomatosis-associated angiomyolipomas, pulmonary sclerosing hemangioma, dysplasia and squamous cell carcinoma of the oral cavity and esophageal squamous cell carcinoma revealed the influential roles of rpS6 either in benign or malignant tumors [15–18]. Further explorations even showed the positive rpS6-dependent cells viabilities in pancreatic and breast cancers [19, 20]. Moreover, mTOR inhibitors, which act as the dephosphorylator of rpS6, are currently being evaluated as antiproliferative drugs in several human malignancies, providing more functional evidence for rpS6 [21, 22]. However, the slower proliferation of mice embryonic cells with the increased phosphorylation of rpS6 was observed as well to contradict the traditional ideas [6].

Regarding the effects of rpS6 in NSCLC, it has been poorly understood so far. In this study, we immunohistochemically detected the expressions of total rpS6 (t-rpS6) and p-rpS6 in NSCLC clinical tissues and analyzed their relevance to the clinical characteristics respectively, establishing p-rpS6 as the activated form of rpS6 in NSCLC. Then, the prognostic values of p-rpS6 in the whole cohort or early staged NSCLC patients were assessed with the Cox regression model. To confirm the clinical findings and investigate the potential mechanisms in which the hyperphosphorylation of rpS6 promotes the development of NSCLC, subsequent cytological experiments with overexpressing or silencing t-rpS6 and p-rpS6 were carried out to observe the changes of cells bioactivities *in vitro*. Moreover, upstream regulation mechanisms of rpS6 were explored as well. Based on the fact that the initiation and progression of NSCLC are largely associated with the exceeding activities of Akt family [23, 24], we attempted to explore whether it relates the functions of rpS6. There are three isoforms (Akt1, Akt2 and Akt3) in the Akt family, and each of them holds distinguishing or even exactly opposite effects in different tumors [25]. Several studies have found that rpS6 is phosphorylated by Akt1(Ser473) approach in colorectal and bladder cancer cells [26, 27], but there are also reports revealing the Akt-independent phosphorylation of rpS6 [7, 28]. Expressions of the three isoforms of Akt and the correlation between Akt and rpS6 phosphorylation, interfered by Akt lentivirus-mediated knockdown or Akt inhibitor XII (Akti-2), were detected separately to determine the principal regulator and signaling pathway of rpS6 in NSCLC.

It is worth mentioning that in accordance with the frequently positive phosphorylation residues of rpS6 in tumors [20, 28, 29], we detected the phosphorylation of Ser235/236 of rpS6 in the present study. Additionally, as no specific phosphorylation inhibitor for rpS6 is commercially available, the small hairpin RNA (shRNA) lentivirus was employed to reduce both of t-tpS6 and p-rpS6. Furthermore, the dephosphorylation of rpS6 was also obtained by Akt shRNA or phosphorylation inhibitor.

## Material and Methods

### Patients and tissue samples

A total of 316 paraffin-embedded archived NSCLC tissue samples and 82 adjacent normal controls were collected from the Department of Pathology, West China Hospital of Sichuan University, China (WCHSU). They were removed from NSCLC patients who had received potentially curative surgeries in WCHSU between 2003 and 2009 without any preoperative therapy. Clinical information was acquired by chart review; whereas the survival data were gotten by telephone interviews, with a mean postoperative follow-up period of 55.9 months. The diagnosis of NSCLC histopathologic determination and differentiation evaluation were determined according to the WHO classification. The International Union against Cancer’s tumor-node-metastasis system was used for pathological staging. All tumors were clinical staged according to the tumor size/node/metastasis (TNM) classification of the IASLC Lung Cancer Staging Project (7th edition) [30]. Clinicopathological characteristics of the 316 cases were summarized in Table 1, while the demographic characteristics comparison of lung cancer patients and controls were displayed in Additional file 1: Table S1. Each patient had signed informed consent for this study, and the ethical approval from the Ethics Committee of Sichuan University was also obtained. All methods were carried out in accordance with the current guidelines and regulations.

**Table 1:** Relationship between clinicopathological characteristics, t-rpS6, p-rpS6 expressions and survival time (*Log-rank*)

Factors		*n* (%)	Median survival time (months)	*Log rank P*
Gender	Male	244 (77.22)	28	0.083
	Female	72 (22.78)	32	
Age/years	<60	130 (41.14)	28	0.942
	≥60	186 (58.86)	31	
Histological type	ADC	142 (44.94)	23	0.146
	SCC	132 (41.77)	32	
	Others	42 (13.29)	22	
Histological differentiation	Poor	150 (47.47)	25	0.008*
	Moderate/Well	166 (50.63)	32	
Tumor size	T1 + T2	167 (52.85)	36	<0.001*
	T3 + T4	149 (47.15)	21	
Lymph node invasion	N0	138 (43.67)	37	<0.001*
	N1 + N2 + N3	178 (56.33)	25	
Distant metastasis	M0	294 (93.04)	31	0.005*
	M1	22 (6.96)	17	
Stage	I	87 (27.53)	56	<0.001*
	II + III + IV	229 (72.47)	22	
t-rpS6	P	262 (82.91)	28	0.230
	N	54 (17.09)	35	
p-rpS6	P	165 (52.22)	20	<0.001*
	N	151 (47.78)	42	
p-rpS6/t-rpS6	≥0.67	188 (59.49)	12	<0.001*
	<0.67	128 (40.51)	48	

*t-rpS6* total rpS6; *p-rpS6* phosphorylation of rpS6; *ADC* adenocarcinoma; *SCC* squamous cell carcinoma; *P* positive expression; *N* negative expression

*: *P* < 0.05

### Immunohistochemistry procedures

Immunohistochemical staining was performed for t-rpS6 and p-rpS6. The procedure was carried out by the standard approach according to the instructions of Envision immunohistochemical staining kit (DAKO) and previous report [31]. Briefly, each 4 um tissue slide was dried at 65 °C overnight. After the deparaffinization in a series of xylene and dehydration with graded alcohols, 0.3 % fresh hydrogen peroxide in methanol was used to block the endogenous peroxidase. Inhibition for nonspecific binding sites was unnecessary with the Envision kit. Antigen retrieval was performed with 1 mM EDTA buffer (pH 8.0) at 95 °C for 90 min, followed by the free cooling to room temperature. Sections were then incubated with primary antibodies of t-rpS6 (CST, #2217, diluted by 1:100) or p-rpS6 (Ser235/236, CST, #4858, diluted by 1:400) in humid chambers at 37 °C for overnight. The next day, slides were washed by phosphate-buffered saline (PBS, pH 7.2–7.4) for the incubation with the secondary antibody (EnVision™ System, K5027, Dako) for 30 min at room temperature. DAB (3-3’-Diaminobenzidine Tetrahydrochloride) was applied for the color development and then removed by distilled water rinsing. After the nucleus staining with hematoxylin, sections were evaluated under a light microscope. The replacement of the primary antibodies with PBS was used as negative controls; while the known colon carcinoma was considered as positive references.

### Evaluation of the immunohistochemical staining

The staining results were read separately by two experienced pathologists without any clinical information. In cases of disagreement, the third senior advisor was turned to for a consensus. The yellow or brown staining in cytoplasm for rpS6 and p-rpS6 was classified as positive, which was quantitatively defined as the previous description [32]. Briefly speaking, proteins expressions were assessed semiquantitatively in 10 randomly selected fields under 200 magnification, with both of the immunohistochemical intensity and positive fraction. Scores for staining intensity was defined as follows: 0 for no appreciable stain, 1 for barely detectable stains, 2 for readily appreciable brown stains and 3 for dark brown stains. At the same time, scores for the positive proportions were: 0 for no tumor cell positively stained, 1 for less than 10 % of tumor cells stained, 2 for 10 to 50 % of tumor cells stained and 3 for more than half of cells stained. Then the intensity and fractions scores were multiplied to get the final results, from 0 to 9. For statistical analysis, scores of 0 ~ 2 were defined as negative/mild expressions, while 3 ~ 9 were positive (3 to 5: moderate, 6 to 9: strong).

For a more accurate evaluating the activation of rpS6, the ratio of p-rpS6 to t-rpS6 (p-rpS6/t-rpS6) was calculated, which were then divided into the high (≥0.67) and low groups (<0.67).

### Preparation of the overexpressed or small hairpin RNA (shRNA) lentiviruses

Plasmids for rpS6 overexpression (oe) and shRNA silence (sh) were purchased from the Genechem, Shanghai, China. The overexpression construct for rpS6, Akt1, Akt2 and Akt3 were developed by subcloning PCR-amplified full-length cDNA into the plasmid. Two sequences for the rpS6-specific shRNA were 5’-GCAGAATATGCTAAACTTT-3’(sh1) and 5’-TGAACGCAAACTTCGTACT-3’ (sh2), and the shRNA for Akt1, Akt2 and Akt3 were 5’-GAGTTTGAGTACCTGAAGC-3’, 5’-GCACAGGTTCTTCCTCAGC-3’ and 5’-TGGACAAAGATGGCCACATA-3’ respectively. Recombinant lentivirus production (including negative controls without any specific target, NC), cells transfection and stable-transfected clones’ selection were conducted as the previous report [33].

### Cell lines and cell culture

Human bronchial epithelial (HBE) cell line and NSCLC cell lines, including SK-MES-1 for squamous carcinoma and H1650 for adenocarcinoma, were purchased from the American Type Culture Collection. The two NSCLC cell lines were cultured in RPMI-1640 medium (Invitrogen, CA) supplemented with 10 % fetal bovine serum, 100 μg/ml penicillin and 100 μg/ml streptomycin; while HBE were maintained in Keratinocyte-serum free medium (Gibco, USA) with 0.05 mg/ml bovine pituitary extract, 5 ng/ml human recombinant EGF, 0.005 mg/ml insulin and 500 ng/ml hydrocortisone according to the manufacture. In the rpS6 activation mechanism experiment, Akt inhibitor XII Isozyme-Selective Akti-2 (Merck, #124029-2MG) was supplied in the cell culture of H1650 and SK-MES-1 with 0.8 μmol/L, 3.2 μmol/L and 12.8 μmol/L separately, and treated for 24, 48, 72 and 96 h to determine the optimum working condition. All cells were incubated in a humidified aseptic condition at 37 °C with 5 % CO_2_.

### Western blotting analysis

Proteins were extracted from cell lysates using protein extraction kit (KeyGEN, China) and the concentration was measured with the BCA Protein Assay Reagent (Thermo, USA). Then proteins were subjected to the 10 % sodium dodecyl sulfate-polyacrylamide gel electrophoresis (SDS-PAGE; Bio-Rad, Hercules, CA) and electrophoretically transferred to the dPVDF membrane (Amersham Pharmacia Biotech, Piscataway, NJ). Specific protein antibodies included anti-total rpS6 (CST, #2217), anti-p-rpS6 (Ser235/236, CST, #4858), anti-p21^Cip1^ (CST, #2947), anti-p27^Kip1^ (CST, #3688), anti-CDK2 (CST, #2546), anti-CDK4 (CST, #12790), anti-Cyclin E (SAB, #29030), anti-cyclin D1 (CST, #2978), anti-p-Rb (Ser780, CST, #3590), anti-Paxillin (CST, #12065), anti-p-Paxillin (Tyr118; CST, #2541), anti-vimentin (CST, #5741), anti-N-cadherin (CST, #4061), anti-E-cadherin (CST, #3195), anti-MMP-9 (CST, #13667), anti-MMP-2 (CST, # 5741), anti-t-Akt1 (CST, #2967), anti-p-Akt1 (Ser473, CST, # 9018), anti-t-Akt2 (CST, #2964), anti-p-Akt2 (Ser474, CST, #8599), anti-t-Akt3 (Biorbyt, #orb6787), anti-p-Akt3 (Ser472, Biorbyt, #orb6790), anti-t-mTOR (CST, #2983), anti-p-mTOR (Ser2448, CST, #5536), anti-t-p70S6K (SAB, #21276) and anti-p-p70S6K (Ser424, SAB, #21276) and anti-β-actin (CST, #4970) as an internal control. All of the above antibodies were diluted by 1:1000, except for the anti-p-rpS6 by 1:2000. Protein bands were analyzed with the Biometrics digitized image software and recorded as integrated densities (ID). Ratios of ID_(each protein)_ to ID_(β-actin)_ were the final results for proteins levels [34].

### Cell proliferation assays

Cell Counting Kit-8 (CCK-8, Dojindo, Japan) was applied for detecting cells proliferation, with the guidance of the product instruction. Briefly, cells were seeded in 96-well plates repeated by six overnight for full adherent. Then at the time points of 24, 48, 72, 96 and 120 h after the initial culture, CCK-8 liquids were added to react with cells for 1 to 2 h. Cells’ absorbance at 450 nm was measured with the microplate reader (Thermo Scientific, USA).

### Cell cycle assays

The distribution of cell cycles was detected with propidium iodide (PI; Sigma-Aldrich, Oakville, ON) staining and flow cytometry analysis. Cells were harvested with EDTA-free trypsin (Invitrogen, CA) and fixed by 70 % cold ethanol over 12 h. Then PI, together with RNase A and Triton X-100, was applied to stain the cells, followed by the flow cytometry detection and analyzed by the CXP software. Modifit LT 3.1 (USA).

### Wound healing assays

The migration of HBE was evaluated by wound healing assays. HBE cells with different treatment were cultured in six-well plates in monolayer and manually scraped with a 200 μl pipette tip. After 12 and 24 h of this wound, images were taken to observe the closure of the wounds. Six parallel lines were randomly drawn to measure the average distance between the cells borders and ratios of the distance at 12 and 24 h to the beginning (0 h) were recorded as the final results [35].

### Transwell assays

Invasion abilities of H1650 and SK-MES-1 cells were analyzed by the transwell assays with 8 μm pore membrane filters chamber kit (Corning, New York, USA) [36]. Briefly, cells were resuspended with serum-free medium and collected within the top chamber. At the same time, the bottom of wells were filled with complete medium containing 10 % FBS. After the incubation over 24 h, some invasive cells penetrated the polycarbonate membranes and the less invasive ones did not, which could be swabbed off by cotton bud. Then 4’6-diamidino-2-phenylindole dye was used to stain the transmembraned cells and they were manually counted under the fluorescence microscope. The average stained cells in 10 randomly selected fields were recorded as the final results for each chamber.

### Statistical analysis

*Chi-square* test was applied to determine the association between the t-rpS6, p-rpS6 expressions and clinicopathological characteristics, and was also employed to compare the demographic characteristics of NSCLC patients and controls, which was a group analysis, rather than a paired comparison. The survival of patients with different clinical factors and t-rpS6, p-rpS6 expressions were analyzed by *Kaplan-Meier* method and the difference was compared with *Log-rank* test. Univariate Cox regression model was used to calculate the hazard ratio (HR) and the multivariate analysis was performed to identify the independent prognostic predictors. Results for the cell proliferation, cell cycles distribution, wound healing, transwell and Western blotting assays were all expressed as mean ± standard deviation (SD) and compared by one-way analysis of variance (ANOVA) with LSD test between any two groups. All statistical analysis was carried out using the software of SPSS 18.0 for Windows (SPSS, Chicago, IL, USA). Differences were considered statistically significant for $P$ value less than 0.05.

## Results

### Both of t-rpS6 and p-rpS6 were highly expressed in NSCLC

**Fig. 1:**
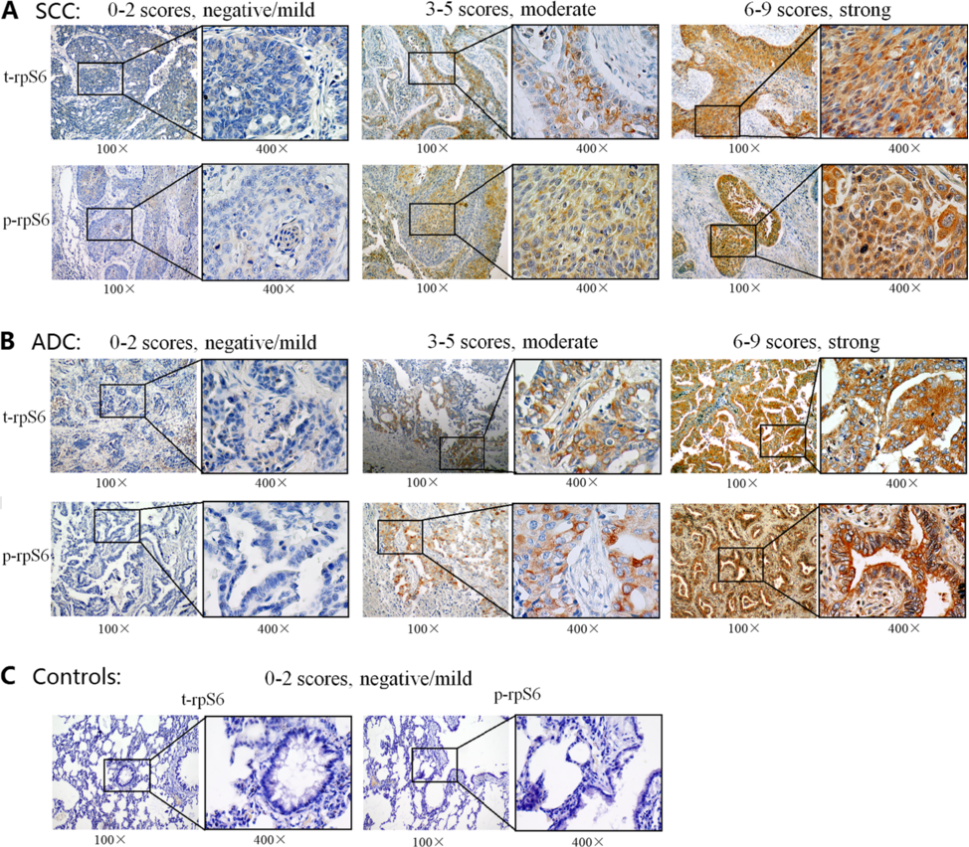
Expression of t-rpS6 and p-rpS6 were both highly expressed in NSCLC tissues. Representative t-rpS6 and p-rpS6 immunohistochemical stainings in squamous cell carcinoma squamous cell carcinoma (**a**) and adenocarcinoma (**b**) tissues showed their abundant accumulations in the cytoplasm of tumor cells, which were divided into negative/mild, moderate and strong grade. However, their expressions in non-tumor lung tissues (**c**) were negative or mild. Each low magnification (100 ×) were paired with a high magnification (400 ×) for clear observation. t-rpS6: total rpS6; p-rpS6: phosphorylation of rpS6; ADC, adenocarcinoma; SCC, squamous cell carcinoma

The expressions of t-rpS6 and p-rpS6 (Ser235/236) were immunohistochemically detected in 316 NSCLC tumor tissues and 82 adjacent normal controls. Demographic characteristics of the NSCLC patients and controls were listed in Additional file 1: Table S1. There was no significant difference in gender, age, smoking or family history of tumors in the two groups (all *P >* 0.05). As shown in Fig. 1, both of t-rpS6 and p-rpS6 were abundantly accumulated in the cytoplasm of NSCLC cells, but less frequently found in non-tumor controls. Of the 316 tumor specimens, 262 cases (82.91 %) were positively stained with t-rpS6 and 165 patients (52.22 %) revealed the p-rpS6 positive expression. In contrast, positive rates for t-rpS6 and p-rpS6 in normal tissues were 62.20 % (51/82) and 21.95 % (18/82) respectively. The results suggested that both of t-rpS6 and p-rpS6 were significantly elevated in NSCLC (both *P <* 0.001).

### Hyperphosphorylation of rpS6, but not the t-rpS6 overexpression, significantly associated with the unfavorable clinical characteristics and survival of NSCLC patients

The relationship between clinical characteristics and t-rpS6, p-rpS6 expressions in the 316 cases of lung cancer patients were summarized in Additional file 1: Table S2. No remarkably different t-rpS6 expression was found in variant clinical characteristics (all *P >* 0.05). However, the hyperphosphorylation of rpS6 was significantly more prevalent in larger tumor size (*P <* 0.001), lymph node invasion (*P <* 0.001), distant metastasis (*P* = 0.015) and advanced stage (*P <* 0.001) patients, whereas the association with sex, age, tumor histological types or histological differentiation was weak (all *P >* 0.05).

Then, the postoperative survival for all patients was analyzed using the *Kaplan-Meier* method and the difference in median survival time was compared with *Log-rank* test. As shown in Table 1 and Additional file 2: Figure S1, poor histological differentiation, enlarged tumors, presence of regional lymph node invasion, distant metastasis and late clinical stage were all greatly correlated with the bad outcome in NSCLC patients (all *P* < 0.05). However, different gender, age or tumor histological types showed slight variation (all *P* > 0.05).

**Fig. 2:**
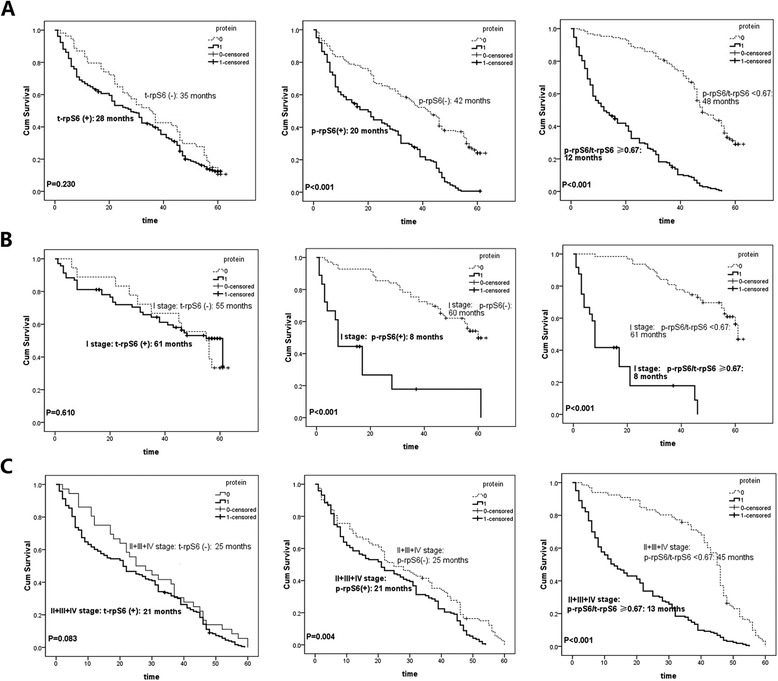
*Kaplan-Meier* survival curves for NSCLC patients with different rpS6 and p-rpS6 expressions. **a** The survival among the whole cohort patients on the basis of t-rpS6, p-rpS6, p-rpS6/t-rpS6 showed the great significance of increased p-rpS6 and elevated p-rpS6/t-rpS6 in NSCLC (both *P* < 0.001) and excluded the role of t-rpS6 overexpression (*P* > 0.05). **b** Survival cures indicated the important prognostic values of the increased p-rpS6 and elevated p-rpS6/t-rpS6 in early staged NSCLC patients (both *P* < 0.001). **c** The hyperphosphorylation of rpS6 also predicted the poor prognosis of advanced staged NSCLC patients (both *P* < 0.05), although the significance was not as remarkable as it in I stage cases. t-rpS6: total rpS6; p-rpS6: phosphorylation of rpS6

Regarding the proteins expressions, elevated t-rpS6 did not predict poor prognosis. The positive t-rpS6 patients held a 5-year survival rate of 11.1 % and the median survival time of 28 months, while the negative ones revealed a 35-month median survival time with 14.9 % cases surviving more than 5 years (Fig. 2a left and Table 1; *P >* 0.05). However, the 5-year survival rate and median survival time of patients with p-rpS6 overexpression were significantly lower than those with negative p-rpS6 (Fig. 2a middle and Table 1; 3.0 % *vs* 26.5 %; 20 months *vs* 42 months, *P* < 0.001). Such results indicated that the overactivation of rpS6, rather than its only overexpression, greatly promoted the progress of NSCLC. For further understanding the effects of rpS6 overactivation, the ratio of p-rpS6 to t-rpS6 (p-rpS6/t-rpS6) was used to report its active extent. Patients with a high p-rpS6/t-rpS6 no less than 0.67 were found to be much more likely to have an adverse prognosis than those with low p-rpS6/t-rpS6 less than 0.67 (Fig. 2a right and Table 1; 2.1 *vs* 32.0 %; 12 months *vs* 48 months, *P* < 0.001).

For supervised analysis including clinical stages, we divided the patients into early (I) and advanced stage (II + III + IV) to explore the influence of rpS6 separately. Not surprisingly, neither the early nor late staged patients showed different survivals with the different t-rpS6 expression (Fig. 2b left and Fig. 2c left; *P* = 0.610 and 0.083, respectively). However, the high p-rpS6 led to poor prognosis both in I stage (Fig. 2b middle; 8 months *vs* 60 months, *P* < 0.001) and advanced patients (Fig. 2c middle; 21 months *vs* 25 months, *P* = 0.004). Similar results were also found for the ratio of p-rpS6/t-rpS6 in different staged patients (Fig. 2b right; 8 months *vs* 61 months, *P* < 0.001 for I stage; Fig. 2c right; 13 months *vs* 45 months, *P* < 0.001 for late stage). It was also easy to find out that rpS6 hyperphosphorylation, reported either the positive expression of p-rpS6 or elevated ratio of p-rpS6/t-rpS6, was a much more significant survival factor for the early staged patients (I stage) than of the late ones (II + III + IV stages) (Fig. 2b middle *vs* Fig. 2c middle; and Fig. 2b right *vs* Fig. 2c right), though all of them revealed statistical significance. These data suggested that p-rpS6 was specifically more relevant to the survival of early staged NSCLC patients.

In the further comparison, an elevated ratio of p-rpS6/t-rpS6 seemed to be a bit more powerful than p-rpS6 alone in predicting the bad outcomes of NSCLC patients (Fig. 2a right *vs* Fig. 2a middle; Fig. 2c right *vs* Fig. 2c middle), despite the weak difference in I stage cases (Fig. 2b right *vs* Fig. 2b middle).

The above results indicated that the hyperphosphorylation of rpS6 was significantly associated with the unfavorable prognosis of NSCLC patients, especially in the early staged cases.

### Hyperphosphorylation of rpS6 was an independent adverse survival marker for NSCLC patients

Based on the findings above, prognostic values of each clinical characteristics and protein expressions were evaluated by the subsequent Cox regression analysis. As shown in Table 2 with univariate assays, risks for bad outcomes in the whole cohort substantially increased with a poor histological differentiation, enlarged tumor size, lymph node invasion, distant metastasis and advanced stage (hazard ratio, HR = 1.369, 2.154, 2.121, 1.835 and 4.143 respectively, all *P* < 0.05), which were completely consistent with the previous *Kaplan-Meier* survival curves. Moreover, patients with a high expression of p-rpS6 or rising p-rpS6/t-rpS6 were also at an increased risk for short survival, especially for the elevated p-rpS6/t-rpS6 (HR = 2.666 and 5.963 respectively with both *P* < 0.001).

**Table 2:** Univariate analysis for the relationship between clinical characteristics, rpS6, p-rpS6 expressions and survival time in different groups

Factors		All patients			Patients in I stage			Patients in II+III+IV stage		
		HR	95 % CI	*P*	HR	95 % CI	*P*	HR	95 % CI	*P*
Gender	Male *vs* Female	1.288	0.961–1.726	0.091	2.021	0.900–4.539	0.088	1.110	0.810–1.522	0.516
Age/years	< 60 *vs* ≥ 60	1.009	0.792–1.286	0.943	1.153	0.639–2.083	0.635	0.906	0.692–1.185	0.469
Histological type	ADC *vs* SCC *vs* others	0.957	0.801–1.144	0.630	0.646	0.397–1.052	0.079	0.975	0.808–1.177	0.793
Histological differentiation	Poor *vs* moderate/well	1.369	1.078–1.174	0.010*	1.604	0.882–2.915	0.122	1.058	0.815–1.374	0.672
Tumor size	T3+T4 *vs* T1+T2	2.154	1.680–2.762	< 0.001*	-	-	-	1.195	0.904–1.508	0.210
Lymph node invasion	N1+N2+N3 *vs* N0	2.121	1.636–2.749	< 0.001*	-	-	-	0.882	0.650–1.197	0.420
Distant metastasis	M1 *vs* M0	1.835	1.181–2.851	0.007*	-	-	-	1.401	0.898–2.185	0.137
Stage	II+III+IV *vs* I	4.143	2.945–5.831	< 0.001*	-	-	-	-	-	-
t-rpS6	P *vs* N	1.206	0.882–1.647	0.241	0.791	0.407–1.536	0.489	1.430	0.989–2.069	0.580
p-rpS6	P *vs* N	2.666	2.056–3.456	< 0.001*	5.916	2.920–11.984	< 0.001*	1.560	1.165–2.089	0.003*
p-rpS6/t-rpS6	≥ 0.67 *vs* < 0.67	5.963	4.437–8.016	< 0.001*	12.304	6.046–25.042	< 0.001*	3.654	2.641–5.056	< 0.001*

*HR* hazard ratio; *CI* confidence interval; *t-rpS6* total rpS6; *p-rpS6* phosphorylation of rpS6; *ADC* adenocarcinoma; *SCC* squamous cell carcinoma; *P* positive expression; *N* negative expression

-: No calculation was carried out because of the absence of dependent factors. For example, tumor sizes in I stage patients were always in T1 or T2, indicating an impossible comparison with T3 and T4 ones. Similarly, patients in I stage were always without any lymph node invasion or distant metastasis

*: *P* < 0.05

Further analysis in different clinical stages was performed as well (Table 2). Only the hyperphosphorylation of rpS6 significantly conferred unfavorable survivals, both in early and advanced staged patients (HR = 5.916 and 12.304 for I stage; HR = 1.560 and 3.654 in late stage; all *P* < 0.001). It was obvious that the significance was much stronger in the early stage as well, especially for the upraised p-rpS6/t-rpS6.

To determine the independent prognostic factors in NSCLC, multivariate Cox regression models were introduced for the statistically significant risk factors in the univariate analysis (Table 3). Because of the similar meaning of p-rpS6 and p-rpS6/t-rpS6, the multivariate assays with one or two of them were performed separately. No matter including either p-rpS6 alone or p-rpS6/t-rpS6 alone, the advanced clinical stage and increased hyperphosphorylation of rpS6 were always independent adverse prognostic factors for NSCLC development (all *P* < 0.05). However, with the two indexes of p-rpS6 and p-rpS6/t-rpS6 combined at the same time, only the increased p-rpS6/t-rpS6 and late clinical stage were found to be the final independent predictors (both *P* < 0.001). Interestingly, results for the p-rpS6/t-rpS6 alone and p-rpS6/t-rpS6 together with p-rpS6 were exactly the same, indicating the dominant significance of p-rpS6/t-rpS6 to p-rpS6.

**Table 3:** Multivariate analysis for the independent risk factors for all patients (Multivariate Cox regression model with the method of forward *LR*)

Factors		HR	95 % CI	*P*
*Including p-rpS6 alone*				
Stage	II + III + IV *vs* I	3.252	2.239–4.723	<0.001*
p-rpS6	P *vs* N	2.403	1.275–2.226	<0.001*
*Including p-rpS6/rpS6 alone*				
Stage	II + III + IV *vs* I	2.377	1.631–3.465	<0.001*
p-rpS6/rpS6	≥0.67 *vs* < 0.67	4.311	3.154–5.894	<0.001*
*Including both of p-rpS6 and p-rpS6/rpS6*				
Stage	II + III + IV *vs* I	2.377	1.631–3.465	<0.001*
p-rpS6/rpS6	≥0.67 *vs* < 0.67	4.311	3.154–5.894	<0.001*

*HR* hazard ratio; *CI* confidence interval; *t-rpS6* total rpS6; *p-rpS6* phosphorylation of rpS6; *P* positive expression; *N* negative expression

*: *P* < 0.05

### Knockdown of t-rpS6, with the synchronous loss of p-rpS6, inhibited the proliferation and invasion of NSCLC cells by the upstream regulation of Akt2 signaling pathway

**Fig. 3:**
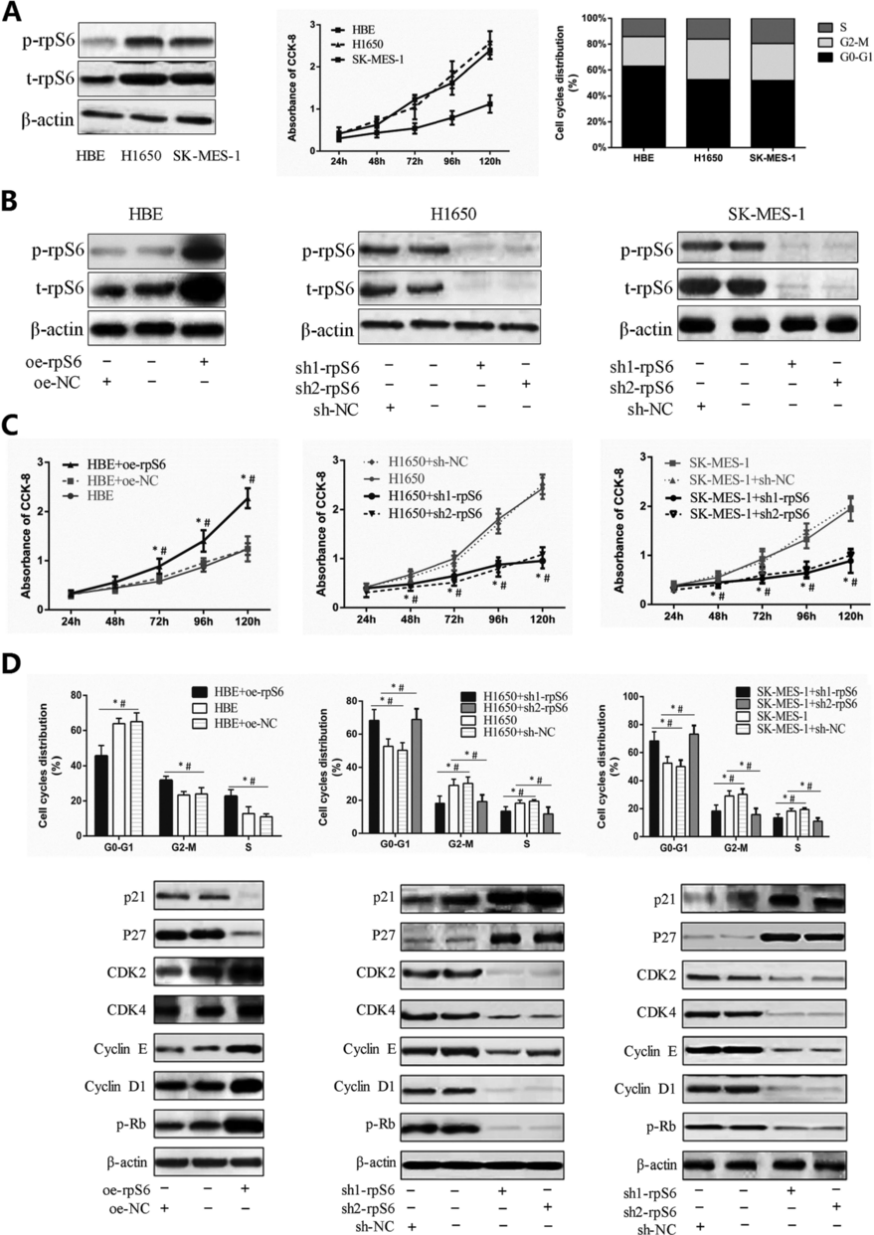
The regulation of rpS6 in cells proliferation. **a** The expressions of t-rpS6 and p-rpS6 in NSCLC cell lines (H1650 for the adenocarcinoma and SK-MES-1 for squamous carcinoma) were also much higher than they were in human bronchial epithelial (HBE) cell lines, (left; all *P* < 0.05). CCK-8 and cell cycle assays showed the active proliferative ability of H1650 and SK-MES-1 (*middle*), and there were more H1650 and SK-MES-1 cells in G2-M0 phase than HBE, but fewer in G2-M phase (*right*). **b** The expressions of t-rpS6 and p-rpS6 in HBE were both significantly increased with the transfection of rpS6 overexpressed lentivirus (oe-rpS6; left) and dramatically decreased in H1650 and SK-MES-1 cell lines with rpS6 knockdown by specific shRNA lentivirus (sh-rpS6; middle and right), separately compared with negative controls (NC). H1650 and SK-MES-1 were both transfected by two different sequences targeting rpS6 (sh1-rpS6 and sh2-rpS6). All *P* < 0.05. **c** After 72 h for the oe-rpS6 transfection, cell proliferation in HBE was significantly increased determined by CCK-8 assays (left). H1650 and SK-MES-1 proliferated much slower after 48 h for the sh-rpS6 transfection (middle and right). All *P* < 0.05. **d** Overexpressions of t-rpS6 and p-rpS6 resulted in more HBE cells entering G2-M and S phase from G0-G1 phase, along with the increase of p-Rb, Cyclin D1, Cyclin E, CDK2, CDK4 and decrease of p21^Cip1^, p27^Kip1^(left). In parallel, loss of t-rpS6 and p-rpS6 induced H1650 and SK-MES-1 G0-G1 arrest and the corresponding expression alterations of cell cycles regulators (middle and right). All *P <* 0.05. The percentage of cells in different phases cells were measured by flow cytometry. CCK-8 experiments were carried out in sextuplicate, and the remaining tests were triplicate. Data are presented as mean ± SD. *: *vs* the corresponding blank control cell lines without any transfection (HBE, H1650 and SK-MES-1 respectively), *P* < 0.05; #: *vs* the corresponding control cell lines with the negative control (NC) vectors transfection (HBE + oe-NC, H1650 + sh-NC, SK-MES-1 + sh-NC, respectively), *P* < 0.05

To identify the potential molecular mechanism by which rpS6 overactivation affects the clinical development of NSCLC, cells biological activities with rpS6 lentivirus mediated interference were analyzed subsequently. Based on the weak expressions of t-rpS6 and p-rpS6 in HBE but strong in the adenocarcinoma cell line of H1650 and squamous cell carcinoma of SK-MES-1 (Fig. 3a left), along with the similarly active characteristics of H1650 and SK-MES-1 (Fig. 3a middle and Fig. 3a right), rpS6 overexpressed or knockdown plasmid was stalely transfected into HBE and H1650, SK-MES-1 individually (Fig. 3b). CCK-8 assay was performed to assess the effects of rpS6 on cells proliferation. Overexpression of t-rpS6, together with the corresponding increase of p-rpS6 (Fig. 3b left), potently promoted the proliferation of HBE cells by comparison with the negative control vectors (NC) and blank HBE (Fig. 3c left; all *P* < 0.05 for the time points of 72, 96 and 120 h after the initial cells seeding). The similar low proliferation of HBE and NC groups excluded the possible off-target effect of the plasmid vector. In contrast, t-rpS6 and p-rpS6 downregulation by shRNA (Fig. 3b middle and Fig. 3b right) significantly attenuated H1650 and SK-MES-1 cells viability compared to the controls, and the two specific shRNA sequences revealed nearly the same effects (Fig. 3c middle and Fig. 3c right; all *P* < 0.001 after 24 h since the cells seeding).

Considering that cell cycles are critical to regulating cells proliferation and viability, the distribution of cell cycles were then detected with PI staining and flow cytometry assays. Results showed that overexpression of rpS6 in HBE led to the remarkable reduction of G1-G0 cells, along with the increase of G2-M and S phase (Fig. 3d left; all *P* < 0.05). Furthermore, sharp elevation of p-Rb, cyclin D1, cyclin E, CDK2 and CDK4 were found with the hyperphosphorylation of rpS6 from the Western blotting assays, whereas p21 and p27, the CDK inhibitors, were dramatically dropped (Fig. 3d left; all *P* < 0.05). Conversely, rpS6 silence and dephosphorylation in H1650 and SK-MES-1 cell lines resulted in the cells predominant aggregation in G0-G1 phase, along with the reduced expressions of p-Rb, cyclin D1, cyclin E, CDK2 and CDK4 but increase in p21 and p27 (Fig. 3d middle and Fig. 3d right; all *P* < 0.05). Representative images of flow cytometry results of cell cycle distribution were placed in the Additional file 3: Figure S2. It was clear that overactivation of rpS6 induced the G0-G1 escape to promote the cells proliferation; while loss of rpS6 attenuated the cells viability by G0-G1 arrest. This might be the one of the potential reasons of the fast progressing of NSCLC patients with rpS6 hyperphosphorylation.

**Fig. 4:**
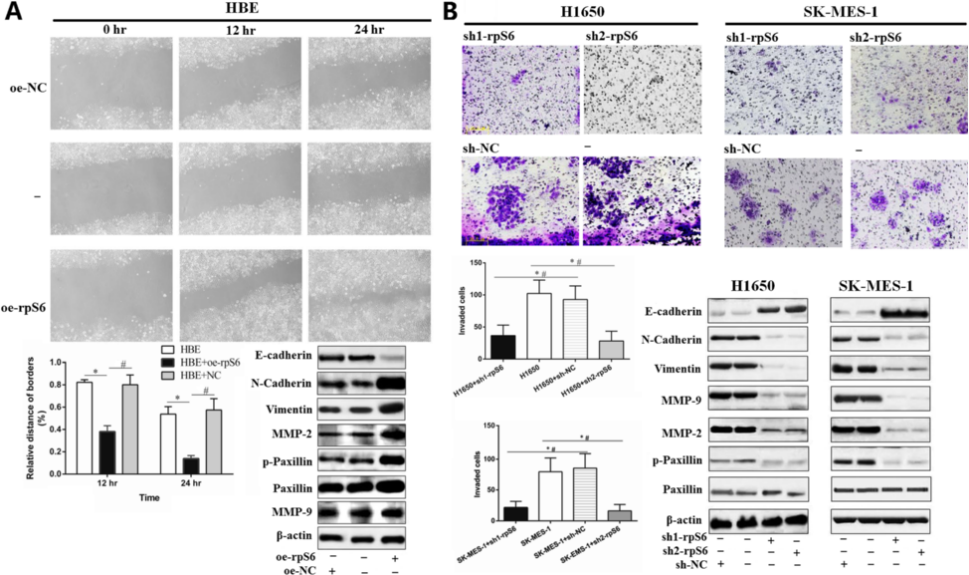
The regulation of rpS6 in cells migration and invasion. **a** Wound healing assays showed that the t-rpS6 and p-rpS6 overexpression in HBE (HBE + oe-rpS6) caused a much faster the healing recover than the two controls (×200). The expressions of N-cadherin, vimentin, MMP-2 and p-Paxillin were remarkably upregulated, but E-cadherin decreased significantly (All *P* < 0.05), in spite of the vague changes of Paxillin and MMP-9 (All *P* > 0.05). **b** Targeted knockdown of t-rpS6 and p-rpS6 (sh1-rpS6 and sh2-rpS6) notably inhibited the metastatic potentials of H1650 and SK-MES-1 cells (×400). The expressions of relative proteins, including E-cadherin, N-cadherin, vimentin, MMP-9, MMP-2 and p-Paxillin changed greatly as well (All *P* < 0.05). All experiments were carried out triplicate. Data are presented as mean ± SD. *: *vs* the corresponding blank control cell lines without any transfection (HBE, H1650 and SK-MES-1 respectively), *P* < 0.05; #: *vs* the corresponding control cell lines with the negative control (NC) vectors transfection (HBE + oe-NC, H1650 + sh-NC, SK-MES-1 + sh-NC, respectively), *P* < 0.05

Given that migration and invasion are critical steps for malignant tumors metastasis, wound healing assay for HBE and transwell experiment for H1650 and SK-MES-1 cell lines were employed to assess the ability of cells migration. As shown in Fig. 4a, overexpression of rpS6 and p-rpS6 prominently enhanced HBE migration. To be specific, the area of the wound in rpS6 activated HBE cells was significantly recovered after 12 h from the gash; and it was almost completely closed after 24 h, which were evidently faster than the two controls (all *P* < 0.05). Subsequent Western blotting assays for the migration relative proteins showed the distinct increase of N-cadherin, vimentin, MMP-2 and p-Paxillin, but reduced E-cadherin in rpS6 hyperphosphorylated HBE cells (Fig. 4a; all *P* < 0.05), though the changes of Paxillin and MMP-9 were almost obscure (Fig. 4a; all *P* > 0.05). Simultaneously, downregulation of t-rpS6 and p-rpS6 in H1650 and SK-MES-1 led to the striking decrease of invasive cells and corresponding alterations of related proteins (Fig. 4b; all *P* < 0.05). All together, these data indicated that rpS6 is involved in the invasion and migration capabilities of NSCLC cells, which might explain the clinical metastasis of NSCLC patients with the abnormal activation of rpS6.

**Fig. 5:**
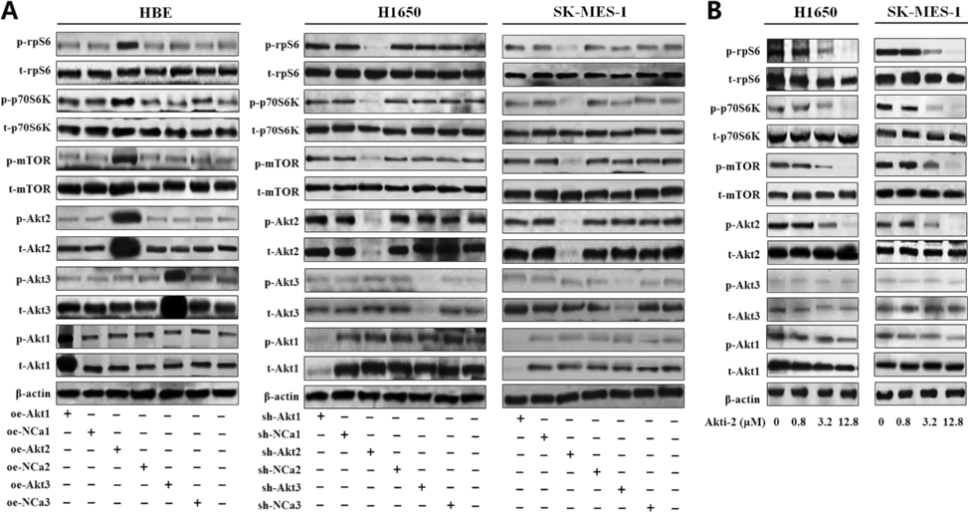
The upstream regulation signaling pathway of rpS6. **a** Separate overexpression of Akt1, Akt2 and Akt3 in HBE cells showed that only Akt2 (oe-Akt2) led to marked hyperphosphorylation of mTOR, p70S6K and rpS6 (left; All *P* < 0.05); while Akt1 or Akt3 alteration resulted in no detectable change of these proteins (left; All *P* > 0.05). In H1650 and SK-MES-1 cell lines, only the specific silence of Akt2 (sh-Akt2) caused significant loss of p-mTOR, p-p70S6K and p-rpS6 (middle and right; All *P* < 0.05), and no notable alteration with the Akt1 and Akt3 knockdown were found (sh-Akt1, sh-Akt3; middle and right; All *P* < 0.05). **b** H1650 and SK-MES-1 were treated with Akti-2 (Akt inhibitor XII) for 72 h in concentration of 0.8, 3.2 and 12.8 μM. Western blot assays revealed the strongest effect of 12.8 μM and the accompanied dephosphorylation of mTOR, p70S6K and rpS6 (All *P* < 0.05). oe-Akt1: overexpression of Akt1; oe-NCa1: negative control for oe-Akt1; oe-Akt2: overexpression of oe-Akt2; oe-NCa2: negative control for oe-Akt2; oe-Akt3: overexpression of oe-Akt3; oe-NCa3: negative control for oe-Akt3. sh-Akt1: knockdown for Akt1; sh-NCa1: negative control for sh-Akt1; sh-Akt2: knockdown for Akt2; sh-NCa2: negative control for sh-Akt2; sh-Akt3: knockdown for Akt3; sh-NCa3: negative control for sh-Akt3

Moreover, regarding the upstream regulation of rpS6, we found that the overexpression of Akt2 in HBE led to dramatical phosphorylation of mTOR, p70S6K and rpS6 (Fig. 5a; all *P* < 0.05), but no decided change was observed with the interference of Akt1 or Akt3 (Fig. 5a; all *P* > 0.05). On the contrary, Akt2 depletion, rather than Akt1 or Akt3, in the two NSCLC cell lines resulted in pronounced dephosphorylation of mTOR, p70S6K and rpS6 (Fig. 5b; all *P* < 0.05 for Akt2), indicating the key role of Akt2 in rpS6 activation. Therefore, the specific Akt inhibitor XII, which is a cell-permeable Akt1/2 inhibitor with improved solubility and Akt2 selectivity (Akti-2), was used for further confirmation. In preliminary experiments, the phosphorylation of Akt2 was detected after incubating with Akti-2 for 24, 48, 72 and 96 h in gradient concentration of 0.8, 3.2 and 12.8 μmol/L. The optimum inhibition was obtained at the point of 72 h. As shown in Fig. 5b, in spite of full incubation for 72 h, neither p-Akt1 nor p-Akt3 was markedly reduced. However, Akt2 were dramatically dephosphorylated in the concentration of 12.8 μmol/L, leading the distinct reduce of p-mTOR, p-p70S6K and p-rpS6. These data displayed the essential regulation role of Akt2/ mTOR/p70S6K signaling pathway in rpS6 activation in NSCLC.

**Fig. 6:**
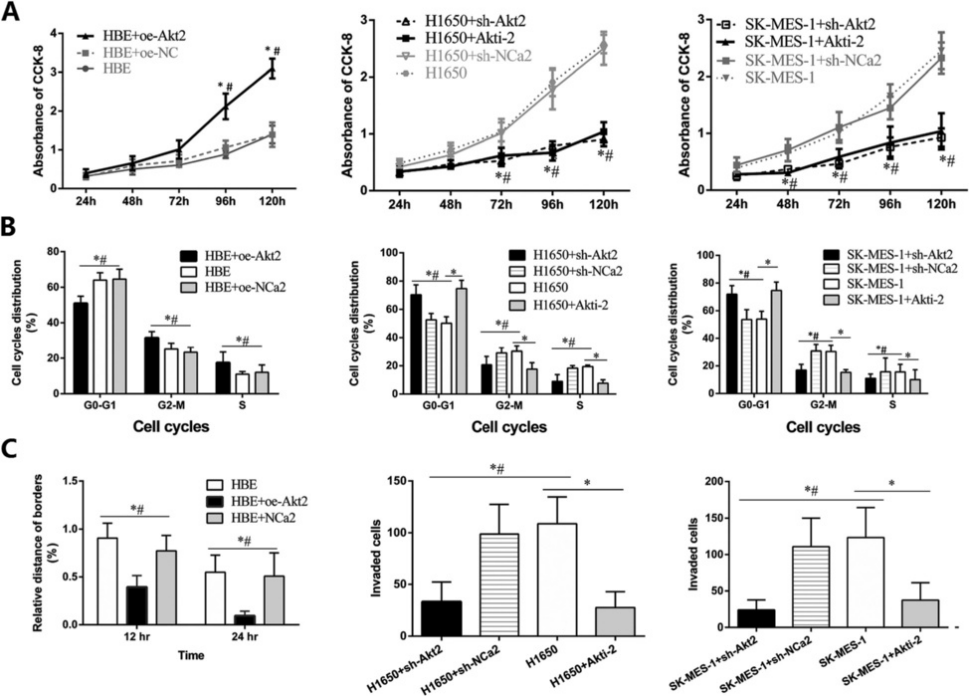
Alteration of cellular bioactivies with the changes of rpS6 phosphorylation from Akt2 interference or phosphorylation inhibition. **a** CCK-8 assay revealed rpS6 hyphosphorylation from Akt2 overexpressing in HBE greatly promoted cells proliferation (left; *P* < 0.05), and p-rpS6 reducing in H1650 and SK-MES-1 cells from Akt2 knockdown or phosphorylation inhibition markedly deteriorated cells proliferation (middle and left; *P* < 0.05). **b** Influence of p-rpS6 on cell cycles in HBE and NSCLC cell lines (*P* < 0.05). **c** Wound-healing assay was performed after rpS6 hyphosphorylation in HBE (left; *P* < 0.05), and cell migration assay was carried out after p-rpS6 reduce in H1650 and SK-MES-1 cell lines (middle and left; *P* < 0.05). CCK-8 experiments were carried out in sextuplicate, and the remaining tests were triplicate. Data are presented as mean ± SD. oe-Akt1: overexpression of Akt1; oe-NCa1: negative control for oe-Akt1; oe-Akt2: overexpression of oe-Akt2; oe-NCa2: negative control for oe-Akt2; oe-Akt3: overexpression of oe-Akt3; oe-NCa3: negative control for oe-Akt3. sh-Akt1: knockdown for Akt1; sh-NCa1: negative control for sh-Akt1; sh-Akt2: knockdown for Akt2; sh-NCa2: negative control for sh-Akt2; sh-Akt3: knockdown for Akt3; sh-NCa3: negative control for sh-Akt3. *: *vs* the corresponding blank control cell lines without any transfection (HBE, H1650 and SK-MES-1 respectively), *P* < 0.05; #: *vs* the corresponding control cell lines with the negative control (NC) vectors transfection (HBE + oe-NC, H1650 + sh-NC, SK-MES-1 + sh-NC, respectively), *P* < 0.05

Biological activities of cell lines with Akt2 overexpression or inhibition were evaluated as well. CCK-8 assays showed that the overactivation of Akt2 in HBE, accompanied by a parallel increase in p-rpS6 expression, but not t-rpS6, had a strong proliferation-promotion effect (Fig. 6a left; *P* < 0.05). However, Akt2 silence by lentivirus or dephosphorylation by Akti-2 markedly deteriorated the phosphorylation of rpS6 and suppressed the proliferation of H1650 and SK-MES-1 cell lines (Fig. 6a middle and Fig. 6 right; all *P* < 0.05). Separate variation of p-rpS6, without any change of t-rpS6, significantly affected the proliferation in all tested cell lines, providing adequate evidence of the crucial employment of rpS6 phosphorylation in NSCLC, rather than its simple overexpression. Analysis of the cell cycle profiles of asynchronous growing cells also revealed the detectable cell cycles redistributions by Akt2 overexpression or inhibition, with the correlate p-rpS6 increasing or reducing (Fig. 6b; all *P* < 0.05). Enhanced migration capability with the high level of p-rpS6 was also observed in HBE by wound healing assays (Fig. 6c left; all *P* < 0.05). In contrast, the number of H1650 and SK-MES-1 cells that were able to invade the pore membrane filter chambers were reduced by nearly 70 % with p-rpS6 inhibition (Fig. 6c middle and Fig. 6c right; all *P* < 0.05). The correlation between rpS6 phosphorylation and cell lines activities, without any change of t-rpS6, might provide full information for the fundamental role of rpS6 hyperphosphorylation in NSCLC, rather than its mere overexpression.

## Discussion

It has been reported that rpS6 has diverse functions in cellular metabolism as well as protein synthesis [6, 37, 38]. Exceptional expression or activation of rpS6 has been found in multiple sclerosis [39], pancreatic cancer [40], squamous cell carcinoma of the oral cavity [17], and even the brain areas with acute ketamine-induced neuroplasticity [41]. In this study, we demonstrate that the expressions of t-rpS6 and p-rpS6 were both significantly abundant in NSCLC tissues. However, only the hyperphosphorylation of rpS6 strongly correlated with the unfavorable clinicopathological characteristics of NSCLC patients and the adverse prognosis. These results indicate that rpS6 is overactivated as phosphorylated, rather than merely overexpressed in NSCLC. Consistent with our results, recent studies also revealed the close relationship between the high level of p-rpS6 and poor survival in esophagus squamous cell carcinoma patients [18], pancreatic neuroendocrine tumors [42] and sarcoma [43]. Clinical investigations even showed the drug resistance with rpS6 hyperphosphorylation in several kinds of malignant tumors [44, 45]. For a more accurate description of the activated degree of rpS6, the ratio of p-rpS6 to t-rpS6 was introduced in our study. As expected, both of the high expression of p-rpS6 alone and the increased ratio of p-rpS6/t-rpS6 were identified as the independent prognostic factors for NSCLC patients. However, further comparison revealed that p-rpS6/t-rpS6 over 0.67 seemed to be a bit more powerful than the p-rpS6 alone. The explanation is not far to seek, because p-rpS6/t-rpS6 as high as 0.67 was only referred to the strong activation of rpS6. Such a combined and quantitative measurement may provide a more accurate method to predict the clinical outcomes of NSCLC patients. In addition, we also found that rpS6 hyperphosphorylation conferred the ominous survival in patients at stage I, which was rather more sensitive than it for the whole or advanced cases; with much more decided survival curves and far bigger HR values. This might be one of the most significant highlights of our study extending the clinical role of p-rpS6 in early staged NSCLC patients. Similar prognostic significance of p-rpS6 was also found in I and II stage esophagus squamous cell carcinoma subjects [18], substantiating the important early predictive values of p-rpS6.

To clarify the potential mechanisms in which p-rpS6 exerts its effects in NSCLC, biological experiments were subsequently conducted. As the specific phosphorylation inhibitor for rpS6 is commercially unavailable, shRNA was used alternatively, which led to the reduced phosphorylation of rpS6 by decreasing the t-rpS6 levels. We found that loss of t-rpS6, together with p-rpS6 downregulation, greatly suppressed the NSCLC cells viability by inducing G0-G1 cell cycles arrest, along with the reduction of CDKs, cyclins and p-Rb. However, rpS6 overexpression and activation in HBE cells had the opposite effects. Similar to our data, the proliferation of several other kinds of cells, including breast carcinoma, cervical carcinoma and even the normal embryonic kidney cell lines, is also dramatically promoted by rpS6 phosphorylation [46]. Liver cells in the late gestation rat revealed p-rpS6 dependent growth as well [47]. Moreover, hepatic cells in the adult mice with conditional deletion of the gene encoding rpS6 failed to proliferate after partial hepatectomy [48]. These results support our findings and suggest that rpS6 phosphorylation plays a crucial role in variant cells proliferation. Regarding the relationship between rpS6 and cell cycle regulation, it was firstly discovered in the fertilized Xenopus eggs with Ras induction nearly 10 years ago [49]. Following studies provided more evidence that the depletion of rpS6 was able to cause p53 induced cell cycles checkpoint impeding or arrest [50, 51], suggesting the possible molecular mechanisms of rpS6 in cell cycles regulation. Recent researches even ascribed the cell cycles regulation effects to the phosphorylation at the site of Ser240/244 [18, 37, 52]. Although our study differed from the reports in the phosphorylated residues, the fact that rpS6-mediated cell viability was largely related to the cells cycles redistributions was almost believable. Continued investigations are required to determine whether the site of Ser235/236 is exclusive phosphorylating target of rpS6 in NSCLC.

Wound healing assay and transwell experiment were performed as well since the cells invasion and motility are critical initiation for tumors spreading and progression. The migration of HBE cell lines distinctly amplified under the induction of rpS6, and this was clearly paralleled by the expressions elevation of p-Paxillin, N-cadherin, vimentin, MMP-2 and reduction of E-cadherin, which are definite regulators of cells adhesion and extracellular matrix degradation. Conversely, H1650 and SK-MES-1 cells migration were dramatically inhibited after rpS6 knockdown. In line with our findings, downregulation of p-rpS6 are also attenuated the cells invasion in esophageal squamous cell carcinoma [18] and renal angiomyolipoma [7]. MJ-56, a new novel anti-metastasis drug, even showed the p-rpS6-mediated invasion inhibition effects in colorectal cancer cells [53]. These results imply that p-rpS6 might be importantly involved in multiple cells invasion and its overexpression will probably facilitate the tumors aggressiveness to a large extent.

Moreover, how is rpS6 activated? Mounting evidence has established the classical way of Akt1(Ser473) in rpS6 activation [26, 27], but nevertheless the Akt-independent phosphorylation of rpS6 was also reported [7, 28]. In evaluating the crosstalk between rpS6 and Akt pathway, we found that the expression of p-rpS6, along with p-mTOR/p-p70S6K, changed markedly with the interference of Akt2 by lentivirus or phosphorylation inhibitor; whereas neither Akt1/p-Akt1 nor Akt3/p-Akt3 led to distinctive effect. The results were supported by the previous study that the decrease of Akt2 reduced the activation of rpS6 in neurons [54].

Responsive cell lines were then employed for bioactivities testing *in vitro*. We found that even though only the p-rpS6 level changed, without any alteration in t-rpS6, cells proliferation, cell cycle distribution and invasion varied in parallel as well. Combined with the clinical findings that only the hyperphosphorylation of rpS6 predicted the unfavorable survival in NSCLC patients, these data provide full evidence that rpS6 abnormal activation as hyperphosphorylation is the key molecule event in the development of NSCLC, and this is probably Akt2 signaling pathway dependent.

## Conclusions

In summary, our study demonstrates that rpS6 is excessively activated in NSCLC as p-rpS6 and this dysregulation is greatly relevant to the clinical progression of NSCLC patients, independently leading an unfavorable prognosis, especially in the early staged cases. Such clinical significance could be confirmed by the cytological bioactivities with the interference of rpS6 activation and it might be derived from the abnormal upstream regulation of Akt2/mTOR/p70S6K signaling pathway. Phosphorylation inhibition of rpS6 may be an effective therapeutic strategy for the treatment of NSCLC.

## Additional files

Additional file 1: Table S1. Demographic characteristics of NSCLC patients and controls (Chi-square test). Table S2. Relationship between clinical characteristics and t-rpS6, p-rpS6 expressions in 316 NSCLC patients. (DOC 76 kb) Additional file 2: Figure S1. The prognostic value of clinical characteristics in NSCLC patients. (TIFF 594 kb) Additional file 3: Figure S2. Representative flow cytometry images of cell cycles tests. (TIFF 1341 kb)

## Abbreviations

rpS6: Ribosomal protein S6
NSCLC: Non-small cell lung cancer
ADC: Adenocarcinomas
SCC: Squamous cell carcinomas
t-tpS6: Total rpS6
p-rpS6: Phosphorylation of rpS6
oe-: Overexpression
shRNA: Small hairpin RNA
sh1-rpS6: shRNA for rpS6 knockdown with the first sequence
sh2-rpS6: shRNA for rpS6 knockdown with the second sequence
NC: Negative control for rpS6
sh-Akt1: shRNA for Akt1 knockdown
sh-Akt2: shRNA for Akt2 knockdown
sh-Akt3: shRNA for Akt3 knockdown
NCa1: Negative control for Akt1
NCa2: Negative control for Akt2
NCa3: Negative control for Akt3. Akti-2, Akt inhibitor XII
