# Supplementary material for: Hyperphosphorylation of ribosomal protein S6 predicts unfavorable clinical survival in non-small cell lung cancer
Source: J Exp Clin Cancer Res. 2015 Oct 21;34:126. doi: 10.1186/s13046-015-0239-1 (PMC4618148; doi:10.1186/s13046-015-0239-1)
Supplement: Additional file 1: Table S1. — Demographic characteristics of NSCLC patients and controls (Chi-square test). Table S2. Relationship between clinical characteristics and t-rpS6, p-rpS6 expressions in 316 NSCLC patients. (DOC 76 kb) [file 13046_2015_239_MOESM1_ESM.doc]

**Supplementary Tables**

| Supplementary Table 1 Demographic characteristics of NSCLC patients and controls (*Chi-square* test) | | | | |
| --- | --- | --- | --- | --- |
| **Factors** | | **Lung Cancer *(n*=316*)***  **(*n*, %)** | **Controls *(n*=82*)***  **(*n*, %)** | ***P*** |
|  |  |  |  |  |
| **Gender** | Male | 244 (77.22) | 65 (79.27) | 0.691 |
|  | Female | 72 (22.78) | 17 (20.73) |  |
| **Age/years** | Median age | 63 | 61 |  |
|  | < 60 | 130 (41.14) | 35 (42.68) | 0.800 |
|  | ≥ 60 | 186 (58.86) | 47 (57.32) |  |
| **Cigarette Consumption** | Never smoking | 32 (10.13) | 8 (9.76) | 0.657 |
|  | Current smokers | 225 (71.20) | 55 (67.07) |  |
|  | Former smokers | 59 (18.67) | 19 (23.17) |  |
| **Family history of tumors** | No | 209 (66.14) | 51 (62.20) |  |
|  | All tumors | 107 (33.86) | 31 (37.80) | 0.238 |
|  | Lung cancer | 46 (14.56) | 6 (7.32) |  |
|  | | | | |

| Supplementary Table 2 Relationship between clinical characteristics and t-rpS6, p-rpS6 expressions  in 316 NSCLC patients | | | | | | | |
| --- | --- | --- | --- | --- | --- | --- | --- |
| **Factors** | |  | **Positive expression of t-rpS6** | |  | **Positive expression of p-rpS6** | |
|  |  |  | ***n* (%)** | ***P*** |  | ***n* (%)** | ***P*** |
| **Gender** | Male (*n*=244) |  | 206 (84.4) | 0.188 |  | 123 (50.4) | 0.237 |
|  | Female (*n*=72) |  | 56 (77.8) |  |  | 42 (58.3) |  |
| **Age/years** | < 60 (*n*=130) |  | 103 (79.2) | 0.146 |  | 68 (52.3) | 0.978 |
|  | ≥ 60 (*n*=186) |  | 159 (85.5) |  |  | 97 (52.2) |  |
| **Histological type** | ADC (*n*=142) |  | 120 (84.5) | 0.787 |  | 75 (52.8) | 0.082 |
|  | SCC (*n*=132) |  | 103 (78.0) |  |  | 62 (47.0) |  |
|  | Others (*n*=42) |  | 29 (69.0) |  |  | 28 (66.7) |  |
| **Histological differentiation** | Poor (*n*=150) |  | 121 (80.7) | 0.314 |  | 86 (57.3) | 0.083 |
|  | Moderate/Well (*n*=166) |  | 141 (84.9) |  |  | 79 (47.6) |  |
| **Tumor size** | T1+T2 (*n*=167) |  | 134 (80.2) | 0.182 |  | 63 (37.7) | **< 0.001*** |
|  | T3+T4 (*n*=149) |  | 128 (85.9) |  |  | 102 (68.5) |  |
| **Lymph node invasion** | N0 (*n*=138) |  | 117 (84.8) | 0.437 |  | 63 (45.7) | **< 0.001*** |
|  | N1+N2+N3 (*n*=178) | | 145 (81.5) |  |  | 102 (57.3) |  |
| **Distant metastasis** | M0 (*n*=294) |  | 245 (83.3) | 0.466 |  | 148 (50.3) | **0.015*** |
|  | M1 (*n*=22) |  | 17 (77.3) |  |  | 17 (77.3) |  |
| **Stage** | I (*n*=87) |  | 69 (79.3) | 0.295 |  | 18 (20.7) | **< 0.001*** |
|  | II+III+IV (*n*=229) |  | 193 (84.3) |  |  | 147 (64.2) |  |
| t-rpS6: total rpS6; p-rpS6: phosphorylation of rpS6; ADC, adenocarcinoma; SCC, squamous cell carcinoma.  *: *P* < 0.05. | | | | | | | |

**Supplementary Figure legends**

**Supplementary Figure 1. The prognostic value of clinical characteristics in NSCLC patients.**

The overall survival curves of gender, age, tumors histological types, differentiation, tumor size, lymph node invasion, distant metastasis, clinical stage in survivals were displayed orderly (a-h).

**Supplementary Figure 2. Representative flow cytometry images of cell cycles tests**

Cell cycles were detected by flow cytometry in HBE with rpS6 overexpression (A), H1650 (B) and SK-MES-1 (C) with rpS6 knockdown.
