# Supplementary figures and images for: Hyperphosphorylation of ribosomal protein S6 predicts unfavorable clinical survival in non-small cell lung cancer
Source: J Exp Clin Cancer Res. 2015 Oct 21;34:126. doi: 10.1186/s13046-015-0239-1 (PMC4618148; doi:10.1186/s13046-015-0239-1)

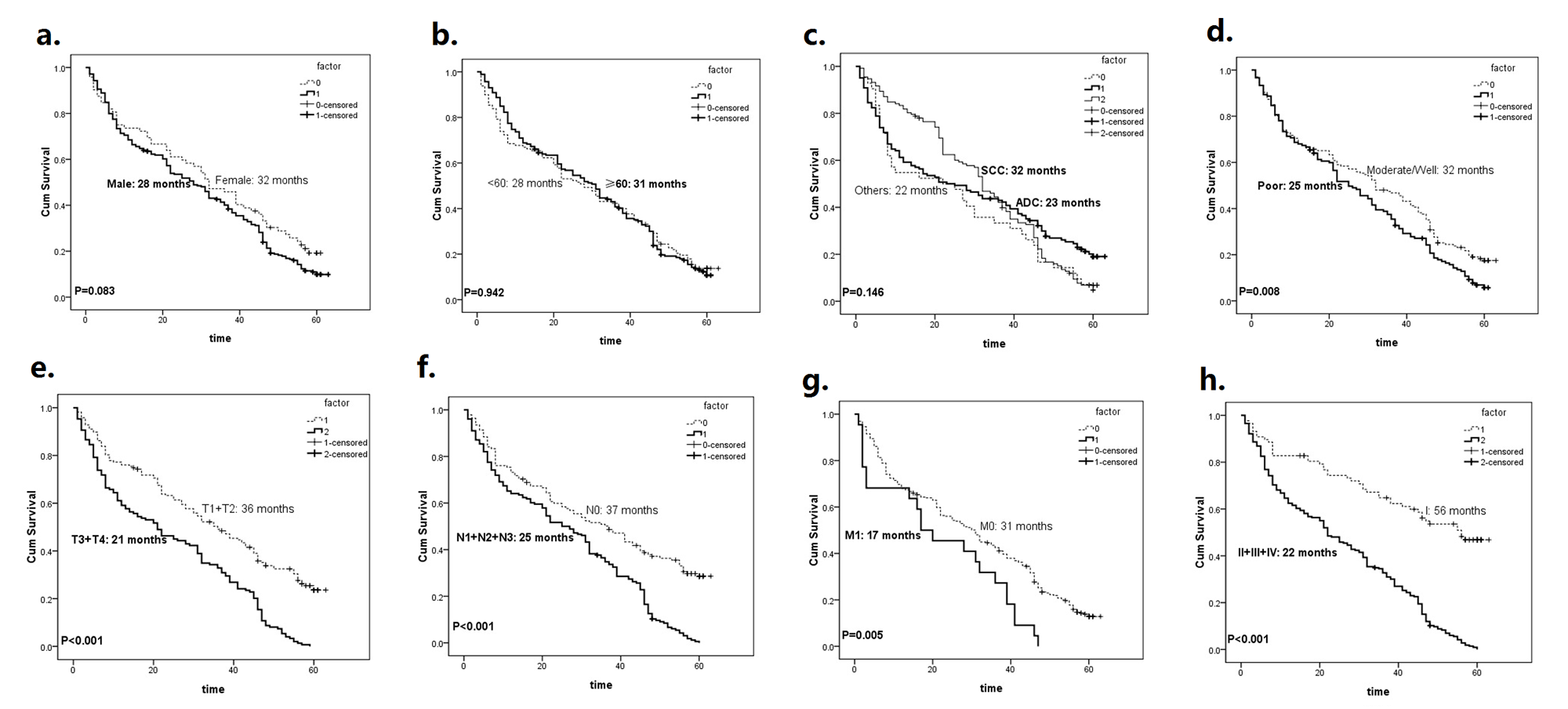

Supplement: Additional file 2: Figure S1. — The prognostic value of clinical characteristics in NSCLC patients. (TIFF 594 kb) [file 13046_2015_239_MOESM2_ESM.tif]

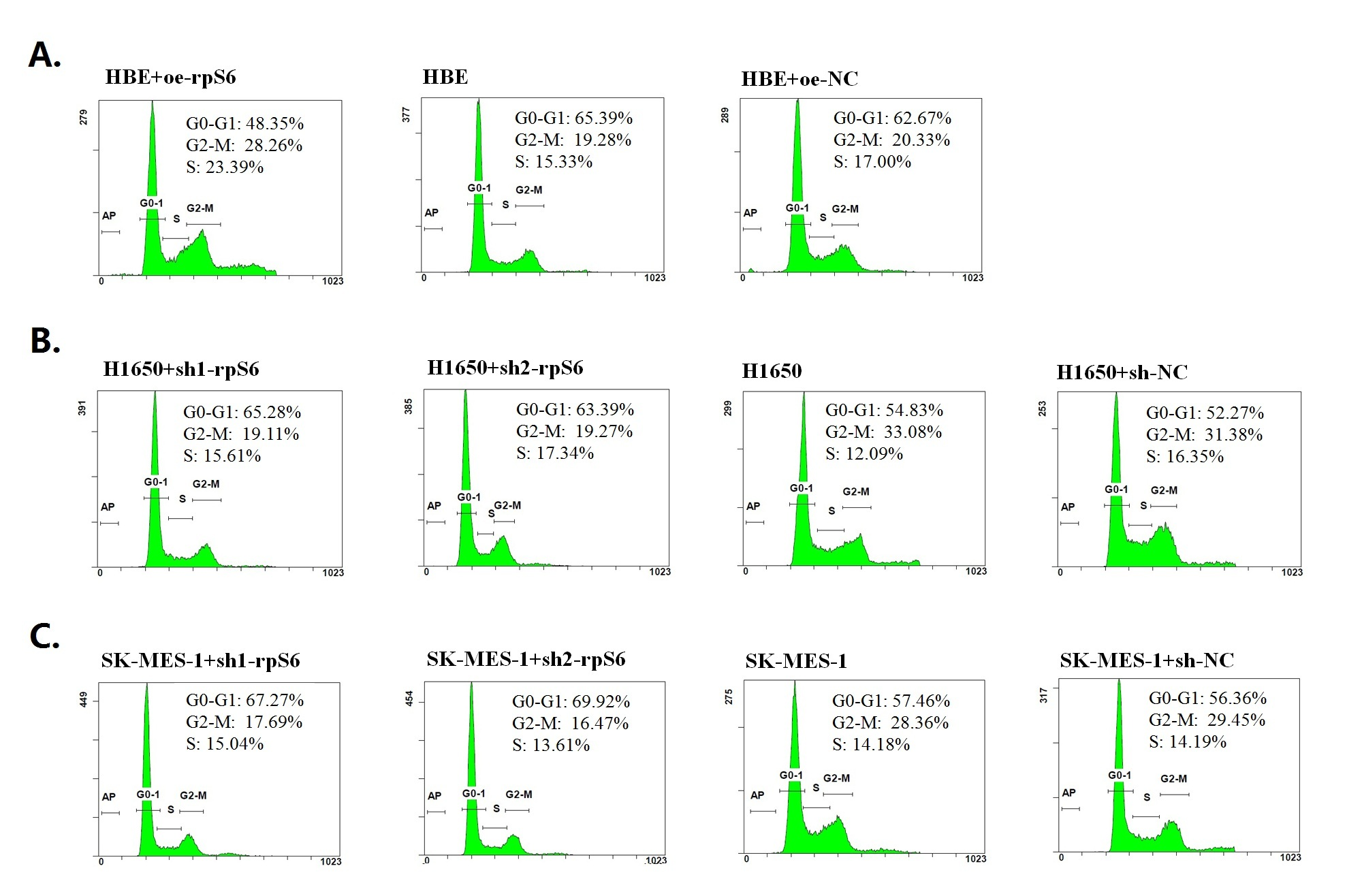

Supplement: Additional file 3: Figure S2. — Representative flow cytometry images of cell cycles tests. (TIFF 1341 kb) [file 13046_2015_239_MOESM3_ESM.tif]
